# Mitochondrial mosaics in the liver of 3 infants with mtDNA defects

**DOI:** 10.1186/1472-6890-9-4

**Published:** 2009-06-05

**Authors:** Frank Roels, Patrick Verloo, François Eyskens, Baudouin François, Sara Seneca, Boel De Paepe, Jean-Jacques Martin, Valerie Meersschaut, Marleen Praet, Emmanuel Scalais, Marc Espeel, Joél Smet, Gert Van Goethem, Rudy Van Coster

**Affiliations:** 1 Department of Pathology, Ghent University Hospital, block A, De Pintelaan 185, 9000 Gent, Belgium; 2 Department of Pediatrics, Division of Pediatric Neurology, Ghent University Hospital, Ghent, Belgium; 3 Metabolic Unit, PCMA, Antwerp, Belgium; 4 Centre Pinocchio CHC Clinique de l'Espérance, Montegnée, Belgium; 5 Center for Medical Genetics, UZ Brussel, Vrije Universiteit Brussel, Brussels, Belgium; 6 Neuropathology, University of Antwerp, Antwerp, Belgium; 7 Radiology and Medical Imaging, Ghent University Hospital, Belgium; 8 Division of Paediatric Neurology, Centre hospitalier de Luxembourg, Luxembourg; 9 Human Anatomy and Embryology, Ghent University, Ghent, Belgium; 10 Division of Neurology and Neuromuscular Reference Center, University Hospital of Antwerp, Antwerp, Belgium

## Abstract

**Background:**

In muscle cytochrome oxidase (COX) negative fibers (mitochondrial mosaics) have often been visualized.

**Methods:**

COX activity staining of liver for light and electron microscopy, muscle stains, blue native gel electrophoresis and activity assays of respiratory chain proteins, their immunolocalisation, mitochondrial and nuclear DNA analysis.

**Results:**

Three unrelated infants showed a mitochondrial mosaic in the liver after staining for COX activity, i.e. hepatocytes with strongly reactive mitochondria were found adjacent to cells with many negative, or barely reactive, mitochondria. Deficiency was most severe in the patient diagnosed with Pearson syndrome. Ragged-red fibers were absent in muscle biopsies of all patients. Enzyme biochemistry was not diagnostic in muscle, fibroblasts and lymphocytes. Blue native gel electrophoresis of liver tissue, but not of muscle, demonstrated a decreased activity of complex IV; in both muscle and liver subcomplexes of complex V were seen. Immunocytochemistry of complex IV confirmed the mosaic pattern in two livers, but not in fibroblasts. MRI of the brain revealed severe white matter cavitation in the Pearson case, but only slight cortical atrophy in the Alpers-Huttenlocher patient, and a normal image in the 3rd. MtDNA in leucocytes showed a common deletion in 50% of the mtDNA molecules of the Pearson patient. In the patient diagnosed with Alpers-Huttenlocher syndrome, mtDNA was depleted for 60% in muscle. In the 3rd patient muscular and hepatic mtDNA was depleted for more than 70%. Mutations in the nuclear encoded gene of *POLG *were subsequently found in both the 2nd and 3rd patients.

**Conclusion:**

Histoenzymatic COX staining of a liver biopsy is fast and yields crucial data about the pathogenesis; it indicates whether mtDNA should be assayed. Each time a mitochondrial disorder is suspected and muscle data are non-diagnostic, a liver biopsy should be recommended. Mosaics are probably more frequent than observed until now. A novel pathogenic mutation in *POLG *is reported.

Tentative explanations for the mitochondrial mosaics are, in one patient, unequal partition of mutated mitochondria during mitoses, and in two others, an interaction between products of several genes required for mtDNA maintenance.

## Background

Mitochondrial heterogeneity after cytochrome oxidase staining has often been visualized in muscle [1-15]. Whether this is caused by varying proportions of mutant and/or depleted versus wildtype mtDNA, has not (completely) been elucidated. Müller-Höcker [16] using COX histochemistry demonstrated a mosaic in the liver of an infant with encephalopathy, cholestatic giant cell hepatitis and mtDNA depletion of unknown origin.

Pearson syndrome (PS) (moderate psychomotor retardation, pancytopenia and pancreatic insufficiency; MIM 557000) and Alpers-Huttenlocher syndrome (AHS) (myoclonal epilepsy, liver and brain disease; MIM 203700) are known to harbour defects of mitochondrial function [17-19], but mitochondrial mosaics in the liver have not been described.

We report on a 2.5 year old girl with PS, a 1-year old boy with AHS, and a 3-year old girl with mtDNA depletion; all show mosaics in their liver parenchyma. In contrast non-parenchymal cells appear microscopically normal.

Partial results were published in abstract form [20].

## Methods

Muscle stains included Gomori-trichrome, fiber typing by ATP-ase after preincubation at pH 4.6, and localisation of COX-and NADH-TR activities according to standard recipes [21]. In the liver cytochrome oxidase activity was visualized with diaminobenzidine according to Seligman et al [22], as modified by Novikoff & Goldfischer [23]. Briefly, liver samples were prefixed in 1% cold buffered glutaraldehyde for 2 hrs in order to preserve ultrastructure. After rinsing, cryostat sections were incubated in open vials at 37° in a DAB medium at pH 6 in acetate buffer containing 0.005 M MnCl_2_, with and without added cytochrome c (1 mg/10 ml) for 2 and 4 hrs. DAB staining of mitochondria was shown to be both O_2 _and cytochrome c dependent [24,25]. For light microscopy (LM) 7 μm sections were mounted in aquamount; for electron microscopy 60 μm sections were postfixed in 1% OsO_4_. Semithin sections were also examined by LM.

Enzymes and metabolites of oxidative phosphorylation were measured in liver, cultured fibroblasts, lymphocytes or muscle.

Blue native PAGE was performed on liver or muscle homogenate as described [26]. MtDNA was analysed by RT-PCR in muscle or leucocytes or liver according to [27]The nuclear gene *POLG *encoding polymerase gamma was sequenced as described [28].

For immunocytochemistry cytospins of cultured fibroblasts were prepared and stained as described [29]. Of liver tissue 8 μm paraffin sections were deparaffinized in xylene and rehydrated in ethanol solutions. After blocking with 2.5% BSA in PBS for 30 min, sections were incubated with primary antibodies in the same solution during 2 hours at room temperature. For the detection of each of the five complexes of the oxidative phosphorylation, monoclonal antibodies were selected that were directed against the gene products of NDUFS7, SDHB, UQCRC2, MTCO1 and ATP5A1 (Invitrogen). Immunodetection was accomplished with the alkaline phosphatase labelled EnVision polymer (Dako) and fast red chromogen. Nuclei were counterstained with hematoxylin and slides were mounted with aquatex.

Ethical issues: all tests and investigations reported in this paper were carried out for diagnostic purposes in the interest of the patients, and under the authority of the university hospitals involved. In particular the parents gave approval for the muscle and liver biopsies, as well as for publication.

## Results

### Patients

**Figure 1:**
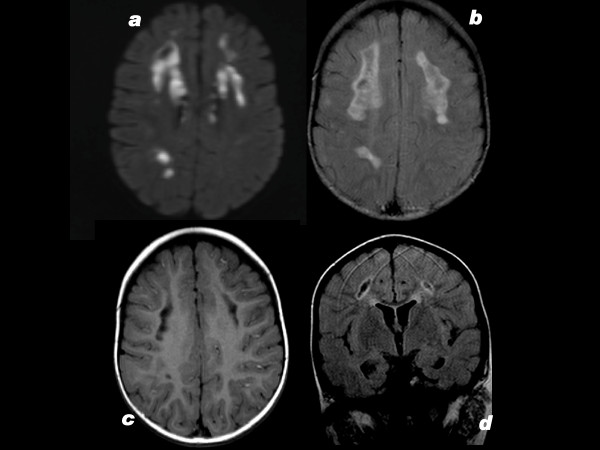
**Patient 1, brain MRI**. 1a: TRACE sequence shows cytotoxic oedema in frontoparietal white matter bilaterally, and in right occipital. 1b: FLAIR sequence, right occipital and bilateral frontoparietal lesions; compare to fig 1a. 1c: 5 months later. T1 weighted without iv contrast. Bifrontal white matter loss with lacunae, small lacuna in right occipital. 1d: Coronal section; bilateral loss of white matter; lacunae lined by high signal interpreted as gliosis.

Patient 1, the daughter of non-consanguineous parents, presents with slight pancytopenia (hemoglobin 9 g/dL, leucocytes 3–5000/ml) and exocrine pancreatic dysfunction (lipase up to 700 U/L but amylase normal). In addition there is moderate psychomotor retardation; at 3 y she is not walking, and talks little. A diagnosis of Pearson syndrome is proposed. MRI shows bilateral cavities in the frontal white matter and right occipital lobe (figures 1a, b, c, d) and absence of the lactate spectrum. Muscle and liver biopsies are performed at 2.5 y. She dies when 3 y 3 m old.

**Figure 2:**
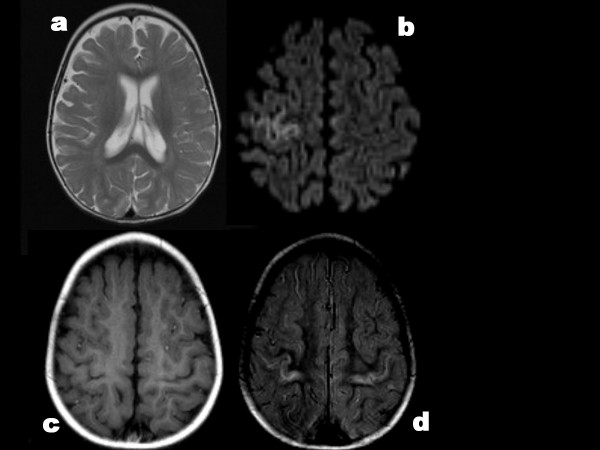
**Patient 2, brain MRI**. 2a: T2 weighted; mild tot moderate dilatation of ventricles and peripheral liquor spaces suggesting cerebral atrophy. 2b: TRACE sequence shows cytotoxic oedema in cortex of right central gyrus. 2c: 3 months later. Focal cortical atrophy of central gyrus bilaterally. 2d: 2 years later. Focal cortical atrophy of central gyrus bilaterally.

Patient 2 is the son of unrelated parents. His uncle suffered from temporal epilepsy. He presents with focal myoclonal epilepsy, axial hypotonia and moderately elevated liver enzymes in the serum (AST 150, 172, 601 U/L; ALT 102, 88, 299 U/L; gGT 83, 93 IU/L). Blood lactate is high. A first MRI at the age of 11 m is normal; but when repeated 4 weeks later it shows slight diffuse cortical atrophy (figures 2a, b, c, d). This leads to the tentative diagnosis of Alpers-Huttenlocher. A muscle biopsy and, at 13 m, a liver biopsy are performed. He dies at 3 y 1 m of age.

Patient 3. Is the daughter of non-consanguineous parents and has normal birthweight. At 3.5 months severe hypoglycemia without ketosis is detected. Hypoglycemia and high blood lactate persist during a fast longer than 5 hrs; it is irresponsive to glucagon. The patient has no cardiomyopathy. Acylcarnitine, sialotransferrine, tyrosinemia, galactosemia and urine organic acids are within normal range. Palmitate and oxanoate oxidation in cultured fibroblasts is normal. Transaminases are increased, and a liver biopsy shows micronodular cirrhosis; glucose-6-phospatase activity is normal. A muscle biopsy and a second liver biopsy are taken at 18 m. MRI is normal at the age of 2 years. At 3 y 9 m, status epilepticus develops which occurs again at 4 y and is difficult to control; and again at 4 y 10 m. At 4 y 11 m there is status epilepticus with myoclonia despite general anaesthesia. She dies from bronchopulmonary infection when 5 y old. The parents agree to an autopsy. This patient is studied in more detail in (Emmanuel Scalais and Baudouin François, in preparation)

### Pathology, biochemistry and molecular biology

Mitochondrial metabolites and respiratory chain activities in muscle or fibroblasts were unremarkable in all three patients. In the liver of patients 1 and 3 complex IV activity was decreased (Additional file 1).

**Figure 3:**
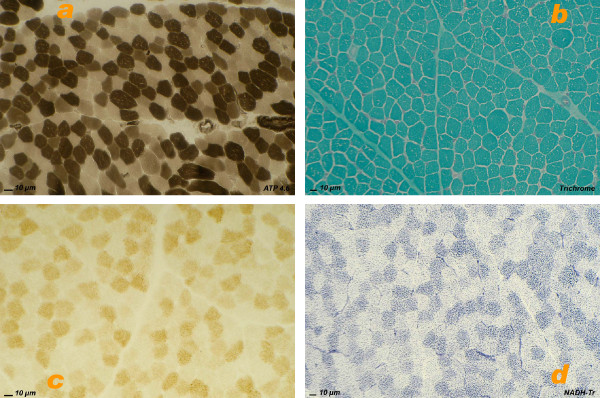
**Muscle pathology**. 3a: ATP-ase reaction at pH 4.6 shows regular sizes and normal chessboard distribution of 3 fiber types. Patient 2. 3b: Gomori trichrome shows normal image; absence of ragged-red fibers. Patient 1. 3c: Cytochrome oxidase activity shows normal distribution of mitochondria in distinct fiber types; compare to 3d. Patient 3. 3d: NADH-tetrazolium reductase shows normal distribution of mitochondria; compare to COX stain in 3c. Patient 1.

Muscles did not show ragged-red fibers; fiber types were normally distributed at random and of comparable sizes (figures 3a, b). COX activity showed normal chess-board distribution of dark type 1 fibers (figure 3c). In patient 2 COX activity seemed diminished, but this can be due to the delay between sampling and staining. Mitochondrial distribution visualized by NADH-TR was always normal (figure 3d).

**Figure 4:**
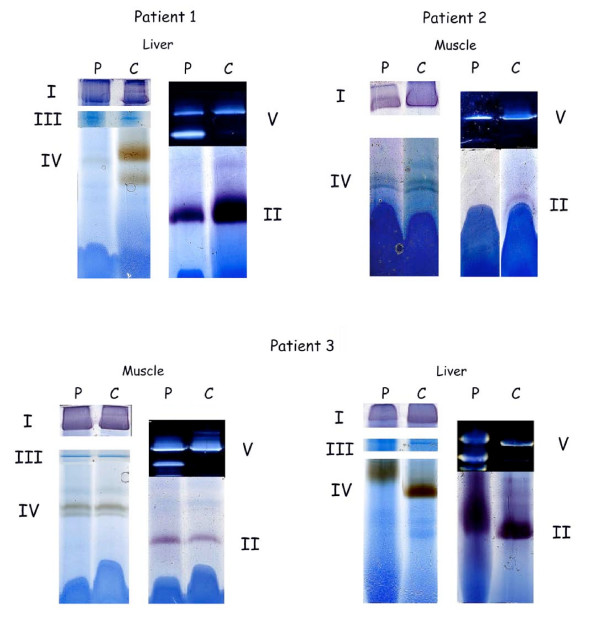
**Blue native PAGE of the OXPHOS complexes**. In-gel activity staining. In patient 1 (Pearson syndrome), the activity of complex IV is severely decreased in liver. In patient 2 (POLG mutation), complex I and complex II are slightly decreased in skeletal muscle (a limited amount of muscle from the patient was available for testing and the complex II band was weak in the control as well). Normal activities are detected in mitochondria from skeletal muscle of patient 3 (POLG mutation). In liver mitochondria of this patient the activities of complex I, III and IV are slightly decreased. In mitochondria from all patients subcomplexes of complex V are seen (only weakly in patient 2), which is considered as a marker of disturbed intramitochondrial protein synthesis.

Blue native PAGE of liver of patient 1 demonstrated very low complex IV (cytochrome oxidase) activity, and a slightly decreased activity in the liver of patient 3, but it was normal in muscle of patients 2 and 3. Complex V (mitochondrial ATP-ase) revealed subcomplexes in patient 1, and in both tissues of patient 3 (figure 4).

#### Liver histology

micro- and macrovesicular steatosis is seen in patients 2 and 3. In patient 2 reticulin lining of hepatocytes was thicker or double, and after CK7 immunoreaction, metaplasia of the parenchyma was detected. Both features suggest a reaction to earlier cell death. However no signs of ongoing necrosis were seen.

**Figure 5:**
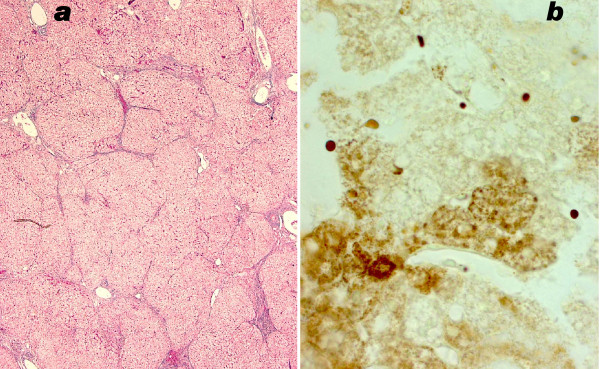
**Liver, patient 3**. 5a: micronodular cirrhosis at 18 months; trichrome stain. 5b: small group of COX reactive parenchymal cells adjacent to an unstained region. Cryostat section, cytochrome oxidase reaction, nuclei slightly counterstained with methylgreen.

In the liver of patient 3 micronodular cirrhosis was obvious, and was even more severe at autopsy (figure 5a). Kupffer cells contained many PAS-positive granules that often were large; they stained positive for acid phosphatase. By electron microscopy as well these lysosomes were conspicuous, but their contents were not suggestive of a specific storage disorder.

#### Cytochrome oxidase localisation in liver

**Figure 6:**
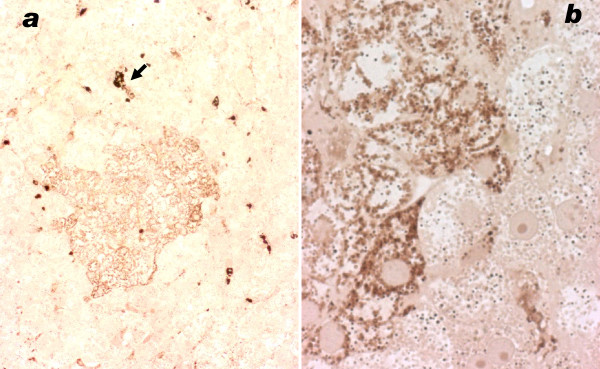
**Liver, patient 1, cytochrome oxidase stain**. 6a: low power view of cryostat section; islet of stained cells surrounded by unstained parenchyma. Large dark granules are erythrocytes in sinusoids and Kupffer cells that stain for peroxidase (arrow). Patient 1. 6b: high power of sharp border between COX stained and unstained parenchymal cells. Numerous tightly packed granules are mitochondria. In the unstained cells, the fewer small round granules are peroxisomes reacting by their catalase. Postosmicated, semithin plastic section.

**Figure 7:**
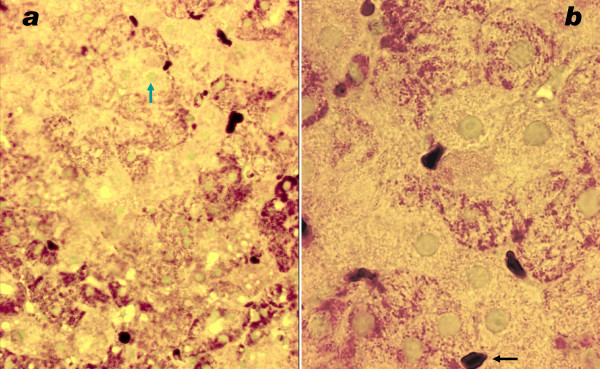
**Liver, patient 2, cytochrome oxidase stain**. 7a: Different levels of mitochondrial staining in adjacent hepatocytes, cryostat section. Nuclei slightly counterstained with methylgreen reveal cells without staining (arrow). 7b: Higher magnification: COX-positive parenchymal cells alternate with unstained ones. Large dark structures are erythrocytes (arrow). Nuclei slightly counterstained with methylgreen, cryostat section.

by LM the DAB staining reaction appeared negative in several liver sections of patients 1 and 3, however in other areas of the biopsy fragments, islets of hepatocytes were found that reacted for COX activity (figures 5b, 6a, b). In patient 2 COX stained regions alternated with weakly staining and unstained ones (figures 7a, b). Sometimes single hepatocytes with stained mitochondria were surrounded by unstained cells. Total area with active COX was estimated to be larger than the unstained areas in pt 2; in contrast COX positive cells were a minority in patients 1 and 3, approximately 10% in patient 3. In patients 2 and 3 bile duct epithelium and arterial smooth muscle cells revealed granular, i.e. mitochondrial staining.

Addition of cytochrome c to the reaction medium did not result in a convincing increase of staining in either patient, indicating that endogenous cytochrome c was not rate-limiting.

**Figure 8:**
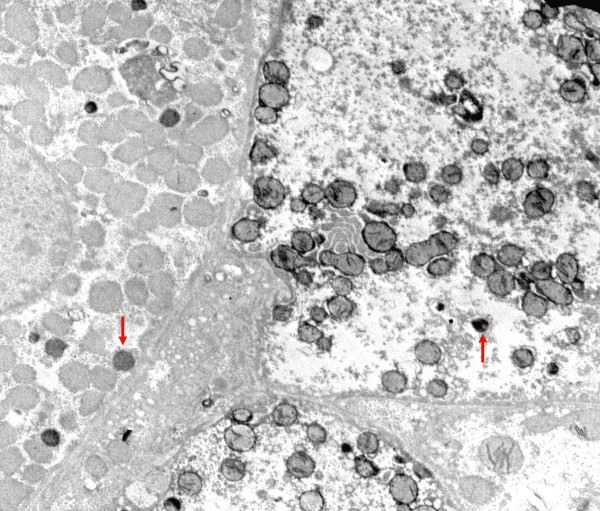
**Liver: ultrastructure and reaction for cytochrome oxidase**. Fig 8: COX reaction product stains mitochondrial envelope and cristae. 4 parenchymal cells have different levels of mitochondrial reaction. Peroxisomes are dark round bodies (arrows), they stand out in the hepatocyte without COX reaction; here mitochondrial numbers appear increased. Patient 1.

**Figure 9:**
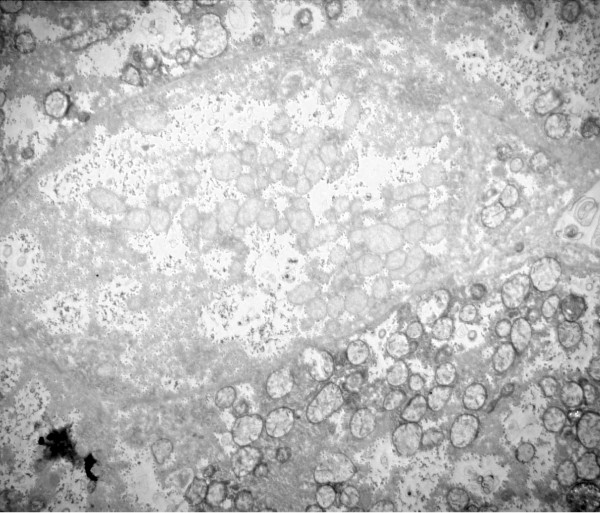
**COX reaction: parenchymal cell in the center shows mitochondria devoid of reaction product**. It is surrounded by cells with well-stained mitochondria. Tiny granules are glycogen. Patient 2.

**Figure 10:**
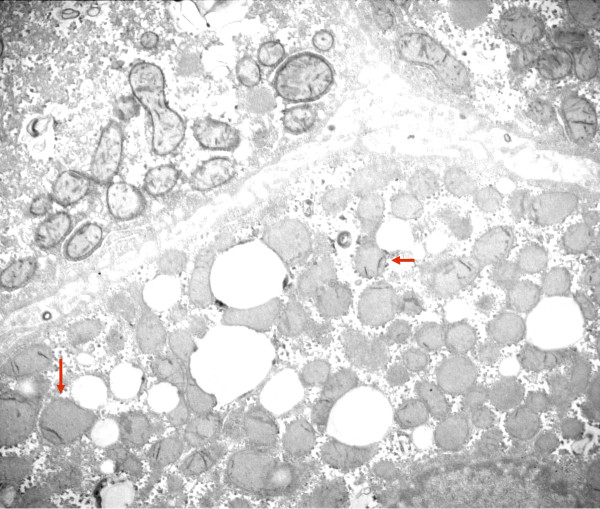
**COX reaction: 2 hepatocytes with well-contrasted mitochondrial envelopes and cristae**. In the third cell that contains many lipid globules, only some mitochondria have a few stained cristae (arrow); mitochondrial numbers appear increased. Patient 2.

**Figure 11:**
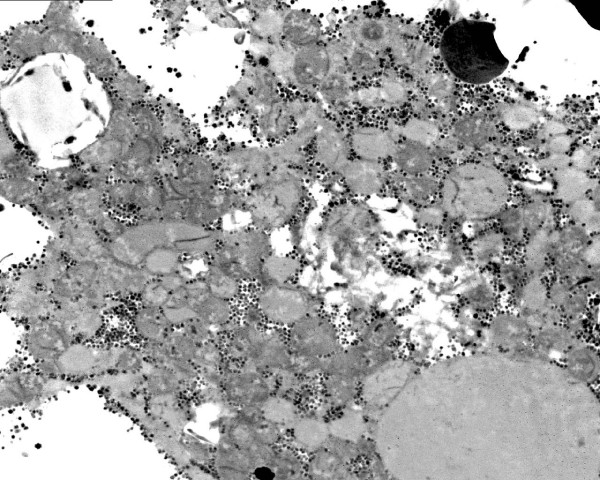
**COX reaction: only single or few cristae show COX reaction product**. Large lipid globules are either dark, grey or largely dissolved. Tiny dark granules are glycogen. Patient 3.

**Figure 12:**
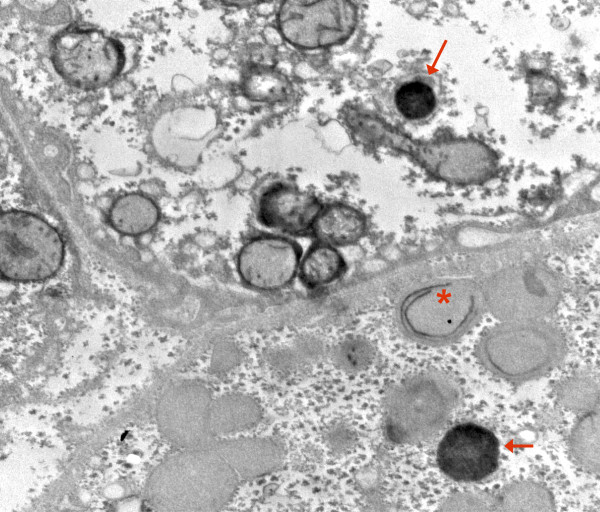
**COX reaction: mitochondrion (asterisk) with only two circular cristae reacting; other mitochondria in this hepatocyte are unstained**. Adjacent cell shows strongly reacting mitochondrial envelopes and cristae; also lipid. Dark bodies are peroxisomes (arrows). Patient 1.

**Figure 13:**
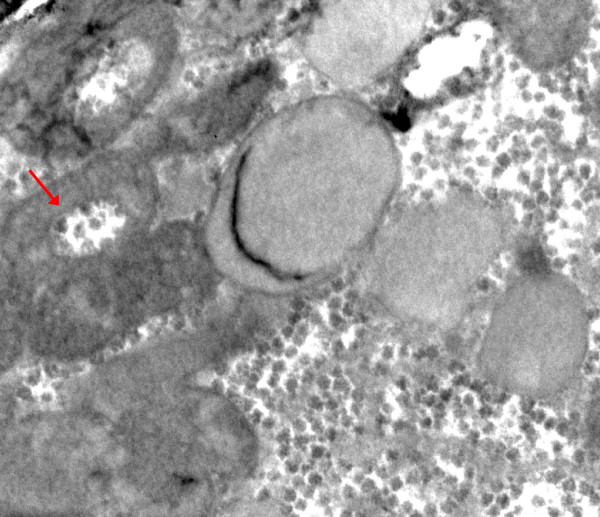
**COX reaction: Single reacting semicircular crista**. Other mitochondria are (nearly) unreactive; two show invaginations (arrow) containing glycogenrosettes and cytosol. Patient 3.

By electron microscopy parenchymal cells with a strong reaction in inner mitochondrial membrane and cristae were found adjacent to cells with mitochondria without visible contrast and mitochondria with one or two cristae that reacted (figures 8, 9, 10, 11, 12, 13). The latter organelles are expected to be invisible by LM. In some of these organelles cristae appeared scarce and/or misshapen (figures 12, 13). In all 3 patients most mitochondria had too few or no cristae by ultrastructural morphology; mitochondrial number seemed to be increased (figures 8, 10) but this was not evident in all hepatocytes. In patient 3 several mitochondria showed invaginations of their envelope containing cytoplasm and glycogen rosettes (figure 13).

**Figure 14:**
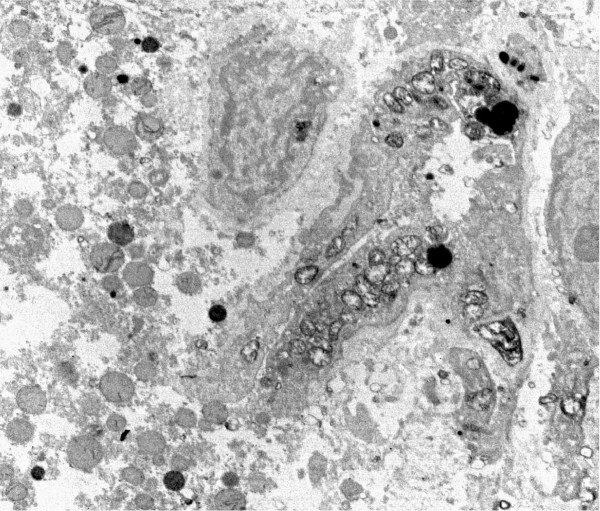
**COX reaction in non-parenchymal cells**. COX reaction: numerous well stained mitochondria in lining cells of a bile duct (or capillary); a basal lamina is present. Adjacent hepatic parenchyma shows dark peroxisomes while mitochondria have only a few reactive cristae. Patient 1.

**Figure 15:**
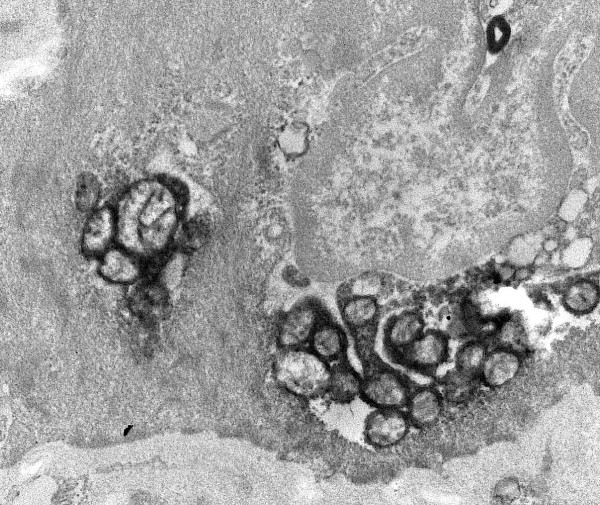
**COX reaction: strongly reactive mitochondria in a cell identified by its microfilaments as vascular smooth muscle**. Patient 1.

In patients 1 and 2 the small mitochondria of bile duct epithelial cells, vascular smooth muscle, endothelial cells, Kupffer cells and neutrophils reacted strongly, adjacent to parenchyma with poorly reactive mitochondria (figures 14, 15). Also in patient 3 positive mitochondria were seen in a non-parenchymal cell.

Glycogen rosettes were abundant in the parenchymal cytosol in the three livers, as is usual in well-fed infants.

Steatosis in the liver of patient 2 was considerable in both hepatocytes with well stained or unstained mitochondria. Since cells with minimal COX reaction showed no ultrastructural signs of anoxia or cell damage, such as dilated ER or swollen mitochondria, a primary mitochondrial enzyme defect is most likely.

#### Immunocytochemistry of OXPHOS proteins

**Figure 16:**
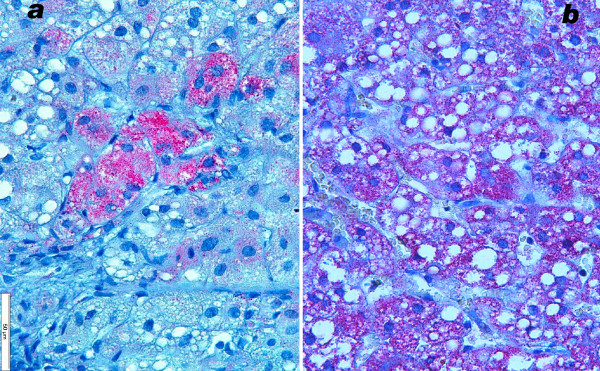
**Immunocytochemistry of OXPHOS proteins in liver of patient 3**. 16a: complex IV (coloured red) is present only in islets of cells amid unstained parenchyma. 16b: complex II is present in red granules, i.e. in mitochondria in all parenchymal cells.

the livers of patients 2 and 3 displayed a mosaic staining pattern for both complexes I and IV (figure 16a). Immunoreactivity for complexes II, III and V was preserved in all liver cells (figure 16b).

In cultured fibroblasts from patient 2, a mosaic of positive and negative cells was observed for complex I; in contrast complexes II, III, IV and V were revealed in all cells. Fibroblasts from patient 3 displayed immunostaining in all cells.

#### DNA analysis

in *patient 1 *(leukocytes) a "common" deletion of 4977 bp in 50% of the mtDNA molecules was found, in agreement with earlier cases of Pearson syndrome.*Patient 2*: muscle showed 60% depletion of the mtDNA content by real time quantitative PCR analysis, and no mutation of mtDNA, in agreement with the clinical diagnosis of Alpers-Huttenlocher syndrome. Subsequent analysis of the nuclear DNA showed a heterozygous recessive mutation in the *POLG *gene, predicting an A467T amino acid substitution [28] in combination with a previously unreported heterozygous recessive splice site mutation c.3643+2t>c.

We also sequenced DNA of both parents, and the father carried the A467T mutation only, whereas the splice site mutation was present in the mother. This finding confirms that both mutations are located on different alleles and suggests that the combination of these mutations is very likely to cause the patients phenotype. Furthermore, the novel mutation alters an evolutionary conserved splice site and is located in the splice donor GT sequence, which is functionally important in the splicing process. Online tools predict that the donor site cannot be recognized as such because of this sequence alteration (e.g. ). We conclude that the c.3643+2T>C mutation is a genuine novel pathogenic mutation in POLG.

*Patient 3*: real time PCR quantification of mtDNA showed a depletion of 74% and 74% of mtDNA contents in muscle and liver, respectively. *POLG *sequencing showed 2 heterozygous recessive mutations, predicting A467T and G848S aminoacid substitutions [28,30].

## Discussion

Although mitochondrial mosaics after COX staining have been known in skeletal muscle and myocardium since 1983, only a single case has been reported in liver [16].

COX histochemistry can be carried out within 24 hrs and is easy to perform. In our patients it yielded evidence, already by light microscopy, of the underlying pathological mechanism, i.e. partial defects in COX activity, whereas tests in blood and muscle were normal or not helpful. The COX deficiency directed subsequent analysis towards blue native electrophoresis of the OXPHOS proteins and to mtDNA.

It is striking that COX activity in skeletal muscle cells, visualized also with DAB, had a normal pattern. Non-parenchymal cells in the liver appeared not to participate in the mosaics, which were limited to the hepatic parenchyma, an observation also made by Müller-Höcker et al [16]. But considering the clinical symptoms it is likely that brain cells, exocrine pancreas and bone marrow had COX deficiency as well. Mild brain atrophy on MRI and psychomotor retardation were also present in the patient of Müller-Höcker et al.

When interpreting the absence of, or very weak stain in single mitochondria at ultrastructure, it should be recalled that the tissue was prefixed in aldehyde which inhibits part of COX activity. One can speculate therefore that liver cells devoid of DAB stain, were actually not entirely without COX activity. Indeed it would be difficult for a cell without any COX to survive for long.

How can the cell- and tissue heterogeneity of the COX activity be explained?

Many disorders have been linked to heteroplasmic alterations in mtDNA: in muscle [6], liver [31], cardiomyocytes and other tissues. Clinical syndromes associated with heteroplasmy are several types of myopathy [5,6]; myoclonus epilepsy and ragged red fibers (MERRF) [4]; progressive external ophtalmoplegia, childhood optic atrophy, followed by deafness and ataxia later [8]; dilated cardiomyopathy [32]; multiple lipomas [33]; Kearns-Sayre syndrome (MIM 530000)[34,35]; focal segmental glomerulosclerosis [36]; early onset diabetes mellitus, optic atrophy and deafness (Wolfram syndrome)[37]; Alpers-Huttenlocher-like disease [31]; and Pearson syndrome with Kearns-Sayre encephalomyopathy [9].

The histochemical COX staining in individual muscle fibers appears to be linked to the expression of a mutation in mtDNA [4] and heterogeneous defects in COX activity may be due to differing populations of wildtype and mutant mtDNA ([10]. Hudson et al [8] showed that COX-positive vs. negative fibers in muscle are correlated to their level of mtDNA deletion.

In *patient 1*, the observed deletion in the mtDNA that encodes for the complex IV protein, might be limited to part of the liver parenchyma *if *this mutation was generated early during liver histogenesis in a single cell hitting only its descendants. But also blood cells bear the mutation (it was found there). There is no simple model for a common origin of both celltypes, blood cells and (part) of the hepatocytes; less so for the neurons and most glia in the brain some of which must have low COX activity.

A different mechanism proposes that the mutation intervened early in development, possibly even in the oocyte, that both mutated as well as wild-type mitochondria proliferated but were distributed at random in the daughter cells, and thus not equally in every cell. This results in varying proportions of normal and defective mitochondria per cell, so-called "shift", or "drift" of the genotype [38]. Larsson et al [35] concluded that "the phenotype can change with time and is governed by fractional concentration in different tissues of the mtDNA with the deletion". Chinnery et al [5] think it is likely that the progression in time of the COX defect in muscle "is due to clonal expansion of mutant mtDNA". The frequently observed sequence in Pearson syndrome of early hematopoietic dysfunction followed later by lactic acidosis, pancreas dysfunction and symptoms of Kearns-Sayre syndrome, has been tentatively explained by "chance distribution of the deleted molecules among the tissues through redistribution" [39]. COX-negative, but also ragged-red muscle fibers (absent in our case) were found in a 8-year old boy who combined the symptoms of Pearson and Kearns-Sayre syndromes [9]. Moreover, we speculate that cell death, ensuing each time COX activity falls below the survival threshold, will trigger compensatory hyperplasia in those tissues that are capable of regeneration, and enable an enrichment with COX-positive cells, i.e. among liver cells of all types, lymphocytes and bone marrow cells, and, in young individuals, even brain cells: glia no doubt, and perhaps even neurons. In neurons proliferative capacity quickly regresses with age; this might explain why the neonate was symptom-free but subsequently developed progressive lesions leading to death.

That cultured fibroblasts of our patients gave normal tests is not unexpected: the phenotypic expression of respiratory chain disorders is unstable in cultured cells and tends to return to normal values [38,40]. In their recent review on disorders of mtDNA synthesis, Freisinger et al. state that fibroblasts are not suitable for assays of depletion [41]. A possible explanation is that cultured cells originate from mothercells that have wild-type mtDNA while the mutated cells die or do not proliferate.

A mosaic of COX activity in muscle has also been observed in a case of a homoplasmic mutation of mtDNA [14]; this is more difficult to explain.

On the other hand *patients 2 and 3 *had a large depletion of the amount of mtDNA.

Depletion syndromes are associated with nuclear mutations of *DGUOK *[42], *SUCLA2*, *SUCLG1*, *TK2*, *MPV17 *[43], *PEO1 *[44], *RRM2B*, and *POLG*; the last is the case in two of our patients. Mutations in the *POLG *gene were previously reported in AHS by three groups [17-19,45-47] and most recently by Stewart et al. [15] who describe 9 patients with a COX mosaic in muscle, but none in liver.

How can one explain a mitochondrial mosaic linked to a nuclear mutation?

Comparing muscle of two patients Moraes et al [48] found a correlation between the degree of mtDNA depletion and COX activity. Since the reduction of mtDNA in cell cultures results in gradual loss of respiratory rate and COX activity [49], the question can be rephrased as follows: why is the level of depletion varying between individual mitochondria, individual cells, and tissues?

Models for tissue specificity of the expression of nuclear mutations impairing the enzymes required for mtDNA synthesis are proposed by several groups; they are based on interactions of distinct gene products required for this synthesis, enzymes as well as metabolites.

*In muscle *the dNTP transporter securing import of cytosolic deoxyribonucleotides for mtDNA synthesis, is normally low. Partial loss by a mutation of the mitochondrial enzyme thymidine kinase-2 will result in critically low levels of DNA. In contrast other celltypes where more dNTP transporter is available will be spared although the mutation is present [50-53].

*In brain *a mutation of succinyl-CoA synthetase-A (required for ADP synthesis) combined to a normally very low mitochondrial NDPKinase (that phosphorylates ADP) will again result in shortage of precursors and thus depletion of DNA [51]. Other tissues have more dNDPK.

The cytosolic deoxycytosinekinase has an overlapping substrate specificity with deoxyguanosinekinase. But in *liver and brain *it is normally very low; a mutation of deoxyguanosinekinase cannot be compensated and will result in DNA depletion limited to those tissues [52] or more severe than in muscle [53]. Vice versa, Taanman et al. have shown that supplementation of dGMP and dAMP to fibroblasts can prevent mtDNA depletion [54]. Hepatic and brain-specific phenotypes are known in mutations of deoxyguanosine kinase [55], and reversal of the phenotype was also described [56].

Sarzi et al. discussing two sibs with a hepatocerebral syndrome and a TWINKLE mutation, suggest that tissue specific differences of nucleotides as well as the normally higher activity of helicase in muscle compared to liver explain the organ specificity of the disease [57].

Galassi et al., discussing a patient with one mutation in POLG-A and a second in ANT1, mention the possibility that the complex clinical features "may be due to cooperative effects consequent to the interrelation of two mitochondrial functions both converging on the homeostatic control of mtDNA maintenance and stability" [58].

Another mechanism for variability in phenotypic expression and possibly for tissue- and cell-specific deficiency is reversal of the mutation in the *TK2 *gene that caused mtDNA depletion [59].

When liver is compared to muscle, in 4 unrelated children with AHS, there was respiratory chain enzyme deficiency in liver, but not in muscle [60]. In a recent series of 10 children with mutations in *POLG1*, 5 had normal enzymes in muscle [61]. The authors conclude that the assay of mitochondrial enzymes in muscle alone is not effective. Mutations in *DGUOK *causing mtDNA depletion nearly always result in normal muscle enzymes, and the study of liver is recommended [41]. The patients with the mutation in *PEO1 *had a severe reduction of OXPHOS complexes in the liver and not in muscle [57]. In a large series of patients with mtDNA depletion and mutations in *DGUOK, POLG*, *MPV17 *or *TK2*, 17 cases had lowered respiratory enzymes in liver but normal activities in muscle [62].

Given the frequency of tissue heterogeneity, mitochondrial mosaics in liver may be more frequent than reported until now.

Whether mosaics after COX staining of the liver were present in these cases from the literature has not been studied.

Finally it is tempting to speculate on the relationship between clinical severity and proportions of COX deficient mitochondria in the mosaics. In patient 1 by far the smallest ratio of stained/unstained mitochondria in the liver coexisted with the severest brain lesions on MRI; on the other hand liver transaminases were unremarkable. *Hepatic *disease was more pronounced in the AH patient, as estimated by the liver enzymes AST, ALT and gGT. The 3rd patient's liver, with 10% of the hepatocytes with reacting mitochondria, had already developed cirrhosis at 3 months of age, but cerebral symptoms appeared only 3 years later. When taking into account the extraordinary regenerative capacity of liver parenchyma, and the near absence of such capacity in the brain, we see as yet no simple correlation between disease severity and the observed distribution of COX deficiency in the liver.

## Conclusion

A tentative explanation for the mitochondrial mosaics is, in patient 1, unequal partition of mutated mitochondria during mitoses, and in patients 2 and 3, an interaction between products of several genes required for mtDNA maintenance.

Histoenzymatic COX staining of a liver biopsy is fast and yields crucial data about the pathogenesis; it indicates whether mtDNA should be assayed. Each time muscle data are non-diagnostic, a liver biopsy should be recommended. Mosaics are probably more frequent than observed until now.

## Competing interests

The authors declare that they have no competing interests.

## Authors' contributions

FR organized the microscopic and histoenzymatic investigations, made the pathological diagnosis of the liver and photographs and drafted the manuscript. PV was the pediatrician in charge of two of the patients, made the clinical diagnosis, and contributed significantly to the manuscript draft. FE is geneticist and was pediatrician in charge of two patients. BF was pediatrician in charge of one patient and contributed to the draft. SS performed the analysis of mtDNA and contributed to the manuscript. BDP carried out and interpreted the immunostaining of OXPHOS proteins. JJM was responsible for muscle pathological diagnosis. VM was responsible for the imaging diagnosis. MP is senior pathologist supervising the examination of the livers. ES was the first pediatrician in charge of one patient. ME contributed to the microscopic diagnosis of one patient. JS carried out and interpreted the blue native electrophoresis and spectrophotometry of OXPHOS enzymes. GVG performed the sequencing of the *POLG *gene and substantially contributed to the manuscript. RVC was pediatrician in charge of the patients, made the clinical diagnosis and conceived and coordinated the mitochondrial investigations and design of the manuscript. All authors approved the manuscript.

## Pre-publication history

The pre-publication history for this paper can be accessed here:



## Supplementary Material

Additional file 1 **Table 1**. OXPHOS activities in patients tissues using spectrophotometry. Click here for file

## References

[B1] Mueller-Hoecker J, Pongratz D, Hubner G (1983). Focal deficiency of cytochrome-c-oxidase in skeletal muscle of patients with progressive external ophtalmoplegia. Cytochemical-fine-structural study. Virchows Archiv A-Pathological Anatomy and Histopathology.

[B2] Mueller-Hoecker J, Johannes A, Droste M, Kadenbach B, Pongratz D, Hubner G (1986). Fatal mitochondrial cardiomyopathy in Kears-Sayre syndrome with deficiency of cytochrome-c-oxidase in cardiac and skeletal muscle. An enzymehistochemical-ultraimmunochemical-fine structural study in muscle in long-frozen autopsy tissue. Virchows Archiv B-Cell Pathology Including Molecular Pathology.

[B3] Oldfors A, Sommerland H, Holme E, Tulinius M, Kristiansson B (1989). Cytochrome-c oxidase deficiency in infancy. Acta Neuropathologica.

[B4] Moslemi AR, Tulinius M, Holme E, Oldfors A (1998). Threshold expression of the tRNA(Lys) A8344G mutation in single muscle fibres. Neuromuscular Disorders.

[B5] Chinnery PF, Howel D, Turnbull DM, Johnson MA (2003). Clinical progression of mitochondrial myopathy is associated with the random accumulation of cytochrome c oxidase negative skeletal muscle fibres. Journal of the Neurological Sciences.

[B6] Karppa M, Herva R, Moslemi AR, Oldfors A, Kakko S, Majamaa K (2005). Spectrum of myopathic findings in 50 patients with the 3243A > G mutation in mitochondrial DNA. Brain.

[B7] Mueller-Hoecker J, Pongratz D, Deufel T, Trijbels JMF, Endres W, Hubner G (1983). Fatal lipid storage myopathy with deficiency of cytochrome-c-oxidase and carnitine. A contribution to the combined cytochemical-fine-structural identification of cytochrome-c oxidase in longterm frozen muscle. Virchows Archiv A-Pathological Anatomy and Histopathology.

[B8] Hudson G, Amati-Bonneau P, Blakely EL, Stewart JD, He LP, Schaefer AM, Griffiths PG, Ahlqvist K, Suomalainen A, Reynier P (2008). Mutation of OPA1 causes dominant optic atrophy with external ophthalmoplegia, ataxia, deafness and multiple mitochondrial DNA deletions: a novel disorder of mtDNA maintenance. Brain.

[B9] McShane MA, Hammans SR, Sweeney M, Holt IJ, Beattie TJ, Brett EM, Harding AE (1991). Pearson syndrome and mitochondrial encephalopathy in a patient with a deletion of mtDNA. American Journal of Human Genetics.

[B10] Matsuoka T, Goto Y, Yoneda M, Nonaka I (1991). MUSCLE HISTOPATHOLOGY IN MYOCLONUS EPILEPSY WITH RAGGED-RED FIBERS (MERRF). Journal of the Neurological Sciences.

[B11] Larsson NG, Tulinius MH, Holme E, Oldfors A, Andersen O, Wahlstrom J, Aasly J (1992). SEGREGATION AND MANIFESTATIONS OF THE MTDNA TRANS RNALYS A-]G(8344) MUTATION OF MYOCLONUS EPILEPSY AND RAGGED-RED FIBERS (MERRF) SYNDROME. American Journal of Human Genetics.

[B12] Oldfors A, Holme E, Tulinius M, Larsson NG (1995). TISSUE DISTRIBUTION AND DISEASE MANIFESTATIONS OF THE TRNA(LYS) A-]G((8344)) MITOCHONDRIAL-DNA MUTATION IN A CASE OF MYOCLONUS EPILEPSY AND RAGGED-RED FIBERS. Acta Neuropathologica.

[B13] Larsson NG, Oldfors A (2000). Mitochondrial myopathies. Acta Physiologica Scandinavica.

[B14] McFarland R, Chinnery PF, Blakely EL, Schaefer AM, Morris AAM, Foster SM, Tuppen HAL, Ramesh V, Dorman PJ, Turnbull M (2007). Homoplasmy, heteroplasmy, and mitochondrial dystonia. Neurology.

[B15] Stewart JD, Tennant S, Powell H, Pyle A, Blakely EL, He L, Hudson G, Roberts M, du Plessis D, Gow D (2009). Novel POLG1 mutations associated with neuromuscular and liver phenotypes in adults and children. J Med Genet.

[B16] Mueller-Hoecker J, Muntau A, Schafer S, Jaksch M, Staudt F, Pongratz D, Taanman JW (2002). Depletion of mitochondrial DNA in the liver of an infant with neonatal giant cell hepatitis. Human Pathology.

[B17] Naviaux RK, Nguyen KV (2004). POLG mutations associated with Alpers' syndrome and mitochondrial DNA depletion. Annals of Neurology.

[B18] Nguyen KV, Ostergaard E, Ravn SH, Balslev T, Danielsen ER, Vardag A, McKiernan PJ, Gray G, Naviaux RK (2005). POLG mutations in Alpers syndrome. Neurology.

[B19] Davidzon G, Mancuso M, Ferraris S, Quinzii C, Hirano M, Peters HL, Kirby D, Thorburn DR, DiMauro S (2005). POLG mutations and Alpers syndrome. Annals of Neurology.

[B20] Roels F, Verloo P, Seneca S, Meersschaut V, Eyskens F, Smet J, Martin JJ, Praet M, Van Coster R (2006). Mitochondrial mosaics in the liver of patients with Pearson and Alpers-Huttenlocher syndromes. Journal of Inherited Metabolic Disease.

[B21] Dubowitz V (1985). Muscle biopsy. A practical approach.

[B22] Seligman AM, Karnovsky MJ, Wasserkrug HL, Hanker JS (1968). Nondroplet ultrastructural demonstration of cytochrome oxidase activity with a polymerizing osmiophilic reagent, diaminobenzidine (DAB). Journal of Cell Biology.

[B23] Novikoff AB, Goldfischer S (1969). Visualisation of peroxisomes (microbodies) and mitochondria with diaminobenzidine. Journal of Histochemistry & Cytochemistry.

[B24] Roels F (1974). Cytochrome-c and cytochrome oxidase in diaminobenzidine staining of mitochondria. J Histochem Cytochem.

[B25] Roels F (1970). Ultrastructural localisation of cytochrome c, in the oocyte of Artemia salina, with 3,3-diaminobenzidine at pH9. Comptes Rendus hebdomadaires des séances de l' Academie des Sciences Serie D.

[B26] Van Coster R, Smet J, George E, De Meirleir L, Seneca S, Van Hove J, Sebire G, Verhelst H, De Bleecker J, Van Vlem B (2001). Blue native polyacrylamide gel electrophoresis: A powerful tool in diagnosis of oxidative phosphorylation defects. Pediatric Research.

[B27] De Vrieze AS, Van Coster R, Smet J, Seneca S, Lovering A, Van Haute LL, Vanopdenbosch LJ, Martin JJ, Ceuterick-de Groote C, Vandecasteele S (2006). Linezolid-induced inhibition of mitochondrial protein synthesis. Clinical Infectious Diseases.

[B28] Van Goethem G, Dermaut B, Lofgren A, Martin JJ, Van Broeckhoven C (2001). Mutation of POLG is associated with progressive external ophthalmoplegia characterized by mtDNA deletions. Nature Genetics.

[B29] De Paepe B, Smet J, Leroy JG, Seneca S, George E, Matthys D, Van Maldergem L, Scalais E, Lissens W, De Meirleir L (2006). Diagnostic value of immunostaining in cultured skin fibroblasts from patients with oxidative phosphorylation defects. Pediatric Research.

[B30] Lamantea E, Tiranti V, Bordoni A, Toscano A, Bono F, Servidei S, Papadimitriou A, Spelbrink H, Silvestri L, Casari G (2002). Mutations of mitochondrial DNA polymerase gamma A are a frequent cause of autosomal dominant or recessive progressive external ophthalmoplegia. Annals of Neurology.

[B31] Uusimaa J, Finnila S, Vainionpaa L, Karppa M, Herva R, Rantala H, Hassinen IE, Majamaa K (2003). A mutation in mitochondrial DNA-encoded cytochrome c oxidase II gene in a child with Alpers-Huttenlocher-like disease. Pediatrics.

[B32] Arbustini E, Diegoli M, Fasani R, Grasso M, Morbini P, Banchieri N, Bellini O, Dal Bello B, Pilotto A, Magrini G (1998). Mitochondrial DNA mutations and mitochondrial abnormalities in dilated cardiomyopathy. American Journal of Pathology.

[B33] Holme E, Larsson NG, Oldfors A, Tulinius M, Sahlin P, Stenman G (1993). Multiple symmetrical lipomas with high levels of mtDNA with the tRNAlysa-]G(8344) mutation as the only manifestation of disease in a carrier of myoclonus epilepsy and ragged-red fibers (MERRF) syndrome. American Journal of Human Genetics.

[B34] McKelvie PA, Morley JB, Byrne E, Marzuki S (1991). MITOCHONDRIAL ENCEPHALOMYOPATHIES – A CORRELATION BETWEEN NEUROPATHOLOGICAL FINDINGS AND DEFECTS IN MITOCHONDRIAL-DNA. Journal of the Neurological Sciences.

[B35] Larsson NG, Holme E, Kristiansson B, Oldfors A, Tulinius M (1990). Progressive increase of the mutated mitochondrial-DNA fraction in Kearns-Sayre syndrome. Pediatric Research.

[B36] Hotta O, Inoue CN, Miyabayashi S, Furuta T, Takeuchi A, Taguma Y (2001). Clinical and pathologic features of focal segmental glomerulosclerosis with mitochondrial tRNA(Leu(UUR)) gene mutation. Kidney International.

[B37] Rötig A, Cormier V, Chatelain P, Francois R, Saudubray JM, Rustin P, Munnich A (1993). Deletion of mitochondrial-DNA in a case of early-onset diabetes-mellitus, optic atrophy, and deafness (Wolfram syndrome, MIM 222300). Journal of Clinical Investigation.

[B38] Munnich Arnold, Rötig Agnes, Cormier-Daire Valérie, Rustin Pierre, Scriver Charles R, Beaudet Arthur L, Sly William S, Valle David, Childs Barton, Kinzler Kenneth W, Bert V (2001). Clinical presentation of respiratory chain deficiency. The Metabolic & Molecular Basis of Inherited Disease.

[B39] Shoffner JM, Scriver Charles R, Beaudet Arthur L, Sly William S, Valle David, Childs Barton, Kinzler Kenneth W, Bert V (2001). Oxidative phosphorylation diseases. The metabolic & molecular basis of inherited disease.

[B40] Heuvel LP van den, Smeitink JA, Rodenburg RJT (2004). Biochemical examination of fibroblasts in the diagnosis and research of oxidative phosphorylation (OXPHOS) defects. Mitochondrion.

[B41] Freisinger PMJ, Rolinski B, Ahting U, Horvath R, Sperl W (2008). Störungen der mitochondrialen DNA-Synthese. Neuropädiatrie in Klinik und Praxis.

[B42] Labarthe F, Dobbelaere D, Devisme L, De Muret A, Jardel C, Taanman JW, Gottrand F, Lombes A (2005). Clinical, biochemical and morphological features of hepatocerebral syndrome with mitochondrial DNA depletion due to deoxyguanosine kinase deficiency. Journal of Hepatology.

[B43] Spinazzola A, Viscomi C, Fernandez-Vizarra E, Carrara F, D'Adamo P, Calvo S, Marsano RM, Donnini C, Weiher H, Strisciuglio P (2006). MPV17 encodes an inner mitochondrial membrane protein and is mutated in infantile hepatic mitochondrial DNA depletion. Nature Genetics.

[B44] Naimi M, Bannwarth S, Procaccio V, Pouget J, Desnuelle C, Pellissier JF, Rötig A, Munnich A, Calvas P, Richelme C (2006). Molecular analysis of ANT1, TWINKLE and POLG in patients with multiple deletions or depletion of mitochondrial DNA by a dHPLC-based assay. European Journal of Human Genetics.

[B45] Naviaux RK, Nyhan WL, Barshop BA, Poulton J, Markusic D, Karpinski NC, Haas RH (1999). Mitochondrial DNA polymerase gamma deficiency and mtDNA depletion in a child with Alpers' syndrome. Annals of Neurology.

[B46] Ferrari G, Lamantea E, Donati A, Filosto M, Briem E, Carrara F, Parini R, Simonati A, Santer R, Zeviani M (2005). Infantile hepatocerebral syndromes associated with mutations in the mitochondrial DNA polymerase-gamma A. Brain.

[B47] Naviaux RK, Nguyen KV (2005). POLG mutations associated with Alpers syndrome and mitochondrial DNA depletion. Annals of Neurology.

[B48] Moraes CT, Shanske S, Tritschler HJ, Aprille JR, Andreetta F, Bonilla E, Schon EA, Dimauro S (1991). mtDNA DEPLETION WITH VARIABLE TISSUE EXPRESSION – A NOVEL GENETIC ABNORMALITY IN MITOCHONDRIAL DISEASES. American Journal of Human Genetics.

[B49] Rocher C, Taanman JW, Pierron D, Faustin B, Benard G, Rossignol R, Malgat M, Pedespan L, Letellier T (2008). Influence of mitochondrial DNA level on cellular energy metabolism: implications for mitochondrial diseases. Journal of Bioenergetics and Biomembranes.

[B50] Saada A, Shaag A, Mandel H, Nevo Y, Eriksson S, Elpeleg O (2001). Mutant mitochondrial thymidine kinase in mitochondrial DNA depletion myopathy. Nature Genetics.

[B51] Elpeleg O, Miller C, Hershkovitz E, Bitner-Glindzicz M, Bondi-Rubenstein G, Rahman S, Pagnamenta A, Eshhar S, Saada A (2005). Deficiency of the ADP-forming succinyl-CoA synthase activity is associated with encephalomyopathy and mitochondrial DNA depletion. American Journal of Human Genetics.

[B52] Mandel H, Szargel R, Labay V, Elpeleg O, Saada A, Shalata A, Anbinder Y, Berkowitz D, Hartman C, Barak M (2001). The deoxyguanosine kinase gene is mutated in individuals with depleted hepatocerebral mitochondrial DNA (vol 29, pg 337, 2001). Nature Genetics.

[B53] Wang LY, Limongelli A, Vila MR, Carrara F, Zeviani M, Eriksson S (2005). Molecular insight into mitochondrial DNA depletion syndrome in two patients with novel mutations in the deoxyguanosine kinase and thymidine kinase 2 genes. Molecular Genetics and Metabolism.

[B54] Taanman JW, Muddle JR, Muntau AC (2003). Mitochondrial DNA depletion can be prevented by dGMP and dAMP supplementation in a resting culture of deoxyguanosine kinase-deficient fibroblasts. Human Molecular Genetics.

[B55] Freisinger P, Futterer N, Lankes E, Gempel K, Berger TM, Spalinger J, Hoerbe A, Schwantes C, Lindner M, Santer R (2006). Hepatocerebral mitochondrial DNA depletion syndrome caused by deoxyguanosine kinase (DGUOK) mutations. Archives of Neurology.

[B56] De Camaret BM, Taanman JW, Padet S, Chassagne M, Mayencon M, Clerc-Renaud P, Mandon G, Zabot MT, Lachaux A, Bozon D (2007). Kinetic properties of mutant deoxyguanosine kinase in a case of reversible hepatic mtDNA depletion. Biochemical Journal.

[B57] Sarzi E, Steffi G, Serre V, Chretein D, Abdethamid S, Arnold M, Johannes NS, Rotig A (2007). Twinkle helicase (PEO1) gene mutation causes mitochondrial DNA depletion. Annals of Neurology.

[B58] Galassi G, Lamantea E, Invernizzi F, Tavani F, Pisano I, Ferrero I, Palmieri L, Zeviani M (2008). Additive effects of POLG1 and ANT1 mutations in a complex encephalomyopathy. Neuromuscular Disorders.

[B59] Vila MR, Segovia-Silvestre T, Gamez J, Marina A, Naini AB, Meseguer A, Lombes A, Bonilla E, DiMauro S, Hirano M (2003). Reversion of mtDNA depletion in a patient with TK2 deficiency. Neurology.

[B60] Gauthier-Villars M, Landrieu P, Cormier-Daire V, Jacquemin E, Chretien D, Rotig A, Rustin P, Munnich A, de Lonlay P (2001). Respiratory chain deficiency in Alpers syndrome. Neuropediatrics.

[B61] Koch JRJ, Mayr JA, Plecko B, Haberlandt E, Karall D, Tscharre A, Schwarz R, Rauter L, Lauffer H, Tegtmayer F, Müller-Felber W, Röschinger W, Bodamer O, Fütterer N, Rolinski B, Freisinger P, Horvath R, Sperl W (2008). Krankheitsverlauf bei 10 kindern mit polymerase-Gamma-Mutationen. Neuropädiatrie in Klinik und Praxis.

[B62] Sarzi E, Bourdon A, Chretien D, Zarhrate M, Corcos J, Slama A, Cormier-Daire V, de Lonlay P, Munnich A, Rotig A (2007). Mitochondrial DNA depletion is a prevalent cause of multiple respiratory chain deficiency in childhood. Journal of Pediatrics.

