# Supplementary material for: Mitochondrial mosaics in the liver of 3 infants with mtDNA defects
Source: BMC Clin Pathol. 2009 Jun 5;9:4. doi: 10.1186/1472-6890-9-4 (PMC2706255; doi:10.1186/1472-6890-9-4)
Supplement: Additional file 1 — Table 1. OXPHOS activities in patients tissues using spectrophotometry. [file 1472-6890-9-4-S1.doc]

# Additional file

## Table 1: OXPHOS activities in patients tissues using spectrophotometry

Abbreviation: CS, citrate synthase.

Specific activity of citrate synthase is expressed as nanomoles of substrate per minute per milligram of protein. All other data are expressed as the logarithm of OXPHOS complex activities divided by the logarithm of citrate synthase activity. Control sample ratios are given as mean ± SD.

## Table 1

| **Patient** | **Tissue** | **ComplexI/CS** | **Complex II/CS** | **Complex II+III/CS** | **Complex III/CS** | **Complex IV/CS** | **Citrate synthase** |
| --- | --- | --- | --- | --- | --- | --- | --- |
|  |  |  |  |  |  |  |  |
| Patient 1 | liver | 0.76 | 1.00 | 0.94 | 0.85 | 0.58 | 233 |
| *controls (n=11)* | *mitochondrial fraction* | *0.69* ± 0.07 | *0.98* ± 0.09 | *0.94* ± 0.07 | *0.91* ± 0.09 | *1.03* ± 0.08 | *482* ± 128 |
|  |  |  |  |  |  |  |  |
| Patient 2 | skeletal muscle | 0.50 | 0.57 | 0.67 | 0.85 | 0.95 | 297 |
| Patient 3 | skeletal muscle | 0.50 | 0.72 | 0.69 | 0.90 | 0.96 | 149 |
| *controls (n=30)* | *muscle homogenate* | *0.60* ± 0.05 | *0.71* ± 0.06 | *0.69* ± 0.07 | *0.74* ± 0.10 | *0.90* ± 0.06 | *214* ± 61 |
|  |  |  |  |  |  |  |  |
| Patient 3 | liver | 0.26 | 0.97 | 0.45 | 0.57 | 0.52 | 122 |
| *controls (n=30)* | *homogenate* | *0.63* ± 0.10 | *1.06* ± 0.07 | *0.72* ± 0.09 | *0.76* ± 0.11 | *0.86* ± 0.07 | *83* ± 31 |
|  |  |  |  |  |  |  |  |
